# Characterization of Resveratrol, Oxyresveratrol, Piceatannol and Roflumilast as Modulators of Phosphodiesterase Activity. Study of Yeast Lifespan

**DOI:** 10.3390/ph13090225

**Published:** 2020-08-30

**Authors:** Adrián Matencio, Francisco García-Carmona, José Manuel López-Nicolás

**Affiliations:** Departamento de Bioquímica y Biología Molecular A, Unidad Docente de Biología, Facultad de Veterinaria, Regional Campus of International Excellence “Campus Mare Nostrum”, Universidad de Murcia, 30003 Murcia, Spain; adrian.matencio@um.es (A.M.); gcarmona@um.es (F.G.-C.)

**Keywords:** lifespan, phosphodiesterase, stilbenes, roflumilast, resveratrol, *Saccharomyces cerevisiae*

## Abstract

Our desire to live longer has led to an ever-increasing number of novel antiaging products. However, few molecules have any real effect and new ones need to be studied before they can be used commercially. In this contribution, activation of the caloric restriction (CR) pathway was studied using different three (resveratrol, oxyresveratrol and piceatannol)—a family with demonstrated bioactivity on phosphodiesterase activity. The high-affinity phosphodiesterase type 2 (PDE2) of *Saccharomyces cerevisiae* was expressed in *Escherichia coli*, purified and characterized. The activity and the inhibitory activity of each stilbene was studied, and the findings were compared in vitro and in silico with those obtained with roflumilast—a human PDE4 inhibitor widely used in chronic obstructive pulmonary diseases. Finally, an in vivo chronological lifespan assay using WT *S. cerevisiae* and ΔPDE2 *S. cerevisiae* strains was carried out. It was demonstrated that stilbenes can modulate yPDE2 activity, increasing the lifespan of the yeast by 18% over a control (in combination with other pathways). In addition, roflumilast increased the lifespan in the WT strain. The findings as a whole would increase the range of lifespan products available and suggest novel uses for approved drugs.

## 1. Introduction

**Figure 1:**
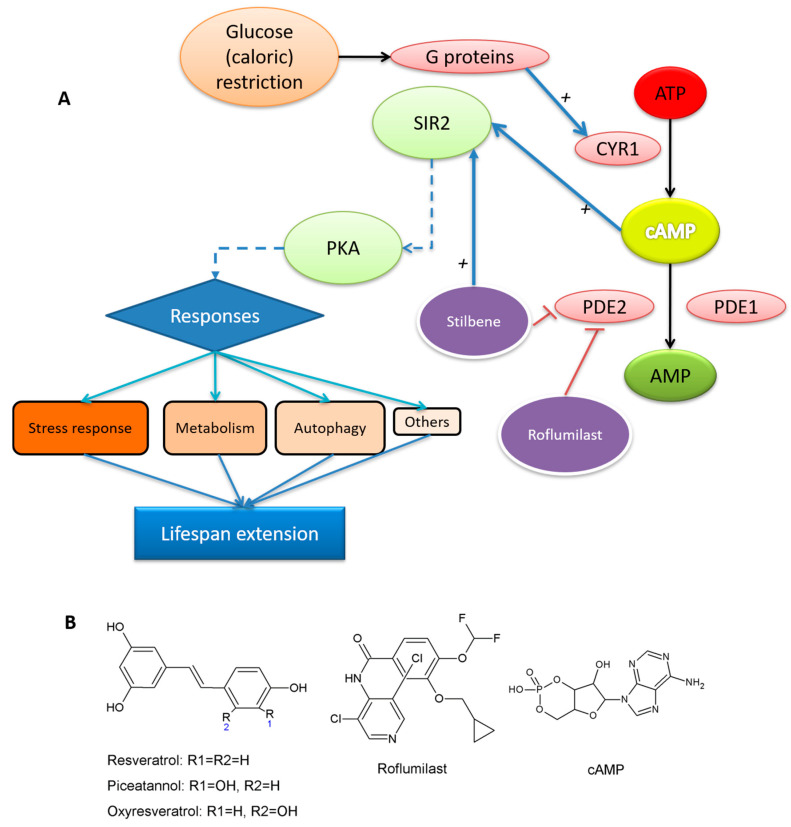
(**A**) Simplified scheme of yeast lifespan extension; (**B**) structure of resveratrol (RSV), oxyresveratrol (OXY), piceatannol (PIC), roflumilast and cAMP.

The life expectancy of citizens is constantly increasing due to greater participation in sports, healthy eating and medical advances. In this respect, the restriction of caloric intake or caloric restriction (CR), has been seen as one of the most effective ways proposed [1,2] of increasing life expectancy. Although this pathway has been studied in several animal models, *Saccharomyces cerevisiae* is one of the most useful models since it is easy to handle and they present several pathways conserved from multicellular eukaryotes [1]. Although there are different ways to manage lifespan [3], the cAMP-PKA signaling mechanism is one of the keys [4,5]. In CR, some molecules and conditions modulate PKA [1,6] by increasing cAMP levels (Figure 1A) suggesting phosphodiesterases (PDEs)—enzymes that convert cAMP into AMP—as possible targets to promote a CR response.

*S. cerevisiae* has two PDEs: PDE1 (cAMP low affinity, Class II; henceforth yPDE1) and PDE2 (high affinity, Class I; henceforth yPDE2) [7,8]. Although both enzymes are able to metabolize cAMP, yPDE2 is more sensitive to cAMP variations [9] and manage cAMP basal levels [10]. Indeed, as a Class I PDE, yPDE2 is more similar to mammalian PDEs [11,12], which is why, this study focuses on the modulation of yPDE2 activity. Recent reviews suggest that the PDE family offers promise for treating diseases such as Alzheimer’s disease, inflammation or erectile dysfunction [13,14,15].

Several authors have reported that yPDE2 knockout has negative effects on lifespan [4,16] because its deletion increases cAMP level in the cells two- to threefold [10]. These data suggest that an excess of cAMP is harmful and yPDE1 is unable to control cAMP. For this reason, the ability to control yPDE2 means of by drugs may represent an interesting strategy to promote the CR response. In this sense, the well-known bioactive compound resveratrol (RSV or RES), a molecule with interesting bioactivities [17], has recently been successfully tested as human PDE inhibitor [18]. Indeed, RSV and chemical derivatives were able to increase the replicative lifespan in *S. cerevisiae* by SIRT1 modulation [19]. Moreover, a recent review about the lifespan extension properties of RSV [20] neatly summarized the possible pathways:

“*Whether SIRT1 is a direct target of resveratrol has been the subject of intense debate. Moreover, resveratrol targets a number of other enzymes, kinases, and receptors. For example, resveratrol inhibits the activity of various cytochrome P450s, enzymes that are involved in phase I drug metabolism and also decreases their transcription* via *inhibition of the aryl hydrocarbon receptor. Quinone reductase 2 (QR2), also involved in drug metabolism, is potently inhibited by resveratrol. In addition to these, resveratrol inhibits the cyclooxygenase enzymes COX-1 and COX-2, resulting in potent anti-inflammatory effects. Finally, resveratrol inhibits a number of important kinases including PKD, which plays an important role in cell proliferation, and S6 kinase, which acts to mediate cell autophagy*.”

These potential bioactivities make stilbenes an interesting family to consider.

For all the above, this work considers the capacity of three stilbenes to inhibit yPDE2: resveratrol, RSV; oxyresveratrol, OXY and piceatannol, PIC (Figure 1B), all known to have important bioactive profiles [17]. Their respective activities were compared with that of roflumilast (Figure 1B), a specific competitive inhibitor of human PDE4 [21] that has been proposed as a CR mimetic preventing diabetic nephropathy [22], so perhaps it affects the lifespan. This drug has also shown promising results against Alzheimer’s disease [23].

In addition, a chronological lifespan study [24] was carried out using BY4741 *S. cerevisiae* (henceforth WT) and BY4741 ΔPDE2 *S. cerevisiae* (as for now ΔPDE2) strains to determine their real capacity to increase lifespan.

Bearing the above in mind, the main objectives of this work are:

(1) To express and characterize yPDE2;
(2) To study the effect of RSV, PIC, OXY and roflumilast on yPDE2 activity;
(3) To clarify the inhibition mechanism using molecular docking approach;
(4) To carry out a chronological lifespan assay with the inhibitors.

## 2. Materials and Methods

### 2.1. Materials

Resveratrol, oxyresveratrol and piceatannol were purchased from TCI Europe and used as received. Roflumilast (CID 5281717) was purchased from Xi An Kerui Biochemical CO (Xi’an, China) and used as received. The samples were stored in darkness. HPLC grade solvents were purchased from Fisher Scientific (Madrid, Spain). The remaining chemicals were purchased from Sigma-Aldrich (Madrid, Spain).

### 2.2. Equipment and Experimental Procedure

#### 2.2.1. Sequence Analysis

The UniProt database was used to obtain the human and yeast PDEs (database version 2019_07). The information about active sites was obtained from UniProt sequence metadata. For alignment analysis, different EBI software packages were used: The multiple alignments were carried out using *MUSCLE* (multiple sequence comparison by log–expectation) with default parameters. The BLASTp was carried out on the NCBI website with default parameters. The phylogenetic tree was built using the Simple Phylogeny option with distance correction. To show the alignments, *Mview* was used with default options. Pairwise sequence alignment was carried out using *EMBOSS matcher* with Blosum62 matrix and default parameters.

#### 2.2.2. yPDE2 Expression and Purification

The sequence of yPDE2 (Uniprot code P06776) was optimized and synthesized by Genscript and cloned in the pET-28(a) vector to yield an N-terminally His6-tagged protein. The final vector was transformed into *E. coli* (strain Rosetta 2, DE3). To express the protein, the cells were grown with Terrific Broth (Fisher Scientific, Waltham, MA, USA) at 37 °C. When the culture reached 1 D.O., ethanol was slowly added (2% w/w final proportion) and the temperature was lowered to 18 °C. After 1 h, the cells were induced with 0.25-mM isopropyl β-D-1-thiogalactopyranoside for 18 h. Finally, the cells were centrifugated at 2500× *g* and 4 °C and then stored at −80 °C for at least two hours.

Cells were dissolved in 5 mL of binding buffer (0.3-M NaCl, 5-mM imidazole, 0.05-M phosphate buffer pH 7.4 with 0.5-mM benzamidine) and lysed by sonication in 5 pulses of 20 s in a *Branson digital sonifier* (Branson Ultrasonic Corporation, Danbury, CT, USA). Then, they were centrifuged at 8000× *g* for 30 min at 4 °C and purified using a His GraviTrap TALON^®^ column (GE Healthcare, Penzberg, Germany) a wash buffer (0.3-M NaCl, 25-mM imidazole, 0.05-M phosphate buffer pH 7.4 with 0.5-mM benzamidine) and elution buffer (0.3-M NaCl, 300-mM imidazole, 0.05-M phosphate buffer pH 7.4 with 0.5-mM benzamidine) following the manual’s instructions. The final solution was concentrated with Amicon^®^ Ultra15 50 KDa to 100 µL and dissolved twice in binding buffer (3 mL) without imidazole to remove salt contaminations, thus obtaining pure yPDE2+Histag. A 10% SDS-PAGE, dyed with Coomassie blue and silver staining (Sigma-Aldrich GE17-1150-01, Darmstadt, Germany), was carried out to check the purify and molecular weight of the final protein. The concentration of yPDE2 was determined by the Bradford assay (Bio-Rad, Hercules, CA, USA) using bovine serum albumin as standard.

#### 2.2.3. yPDE2 Activity Assay

The assay of yPDE2 activity was carried out as previously described [25]: Briefly, the desired cAMP concentration at a fixed yPDE2 concentration of 0.08 μM (all the assays were carried out with the same preparation of recombinant enzyme) in sample buffer [9], composed of 5-mM Mg_2_SO_4_ 0.1 M Tris-HCl pH 7, were used to directly inject 15 μL into an Agilent 1100 series HPLC system (Santa Clara, CA, USA) and a 1200 series module UV-vis detector with a Kromasil Hydro 150 C18 column (150 mm × 4.6 mm, 5-µm particle size) equipped with an Optiguard^®^ C18 precolumn (Supelco, Darmstadt, Germany) to prevent proteins or contaminants from entering. The conditions were established in the following gradient: solvents: A, Milli-Q water with 0.1% acetic acid and solvent B, 85/15 MeOH/THF *w/w* with 0.1% acetic acid. Conditions: 0–3 min, 0% B at 1 mL/min; 3–12.5 min, 5% B at 2 mL/min; 12.5–17 min, 50% B at 2-mL/min and 17–20 min, 0% B at 2-mL/min all at 30 °C.

To determine K_m_, V_max_ and k_cat_ (product generated per enzyme and time), a nonlinear plot using the Michaelis–Menten kinetic was used.

(1) $$V=\frac{V_{\max}\left[S\right]}{K_{m}+\left[S\right]}=\frac{k_{\mathrm{cat}}\left[E\right]\left[S\right]}{K_{m}+\left[S\right]}$$

where [S] is the substrate concentration and [E] the enzyme concentration.

For the inhibition assay, the type of inhibition was illustrated using the Lineweaver–Burk plot;

(2) $$\frac{1}{V}=\frac{K_{m}}{V_{\max}}\cdot \frac{1}{\left[S\right]}+\frac{1}{V_{\max}}$$

To obtain K_i_, two equations were used: (i) a Michaelis–Menten transformation for competitive inhibition:

(3) $$\frac{K_{m}^{\mathrm{app}}}{K_{m}}=1+\frac{\left[I\right]}{K_{i}}$$

and (ii) the conversion from I_50_ to K_i_ for competitive inhibition [26].

(4) $$K_{i}=\frac{I_{50}}{1+\frac{\left[S\right]}{K_{m}}}$$

#### 2.2.4. Molecular Modeling and Docking

The sequence reported by Genscript for yPDE2 after optimization was uploaded to the Swiss-model [27] with default parameters using PDB ID 3ECN as template. The resulting protein was used to carry out the molecular docking experiments. Our model and the ligand (cAMP, RSV, OXY, PIC and roflumilast; all obtained from the ZINC database) were uploaded to Swiss-Dock [28] with default parameters. For PDE1, the PDB ID 4OJV crystal was used. Only the active site interactions were studied. The results were analyzed using Chimera (version 1.9) and Pymol (version 1.9).

#### 2.2.5. In Vivo Lifespan Study

The in vivo lifespan test was carried out as reported [24] for chronological lifespan (CSL) with slight modifications. The BY4741 “WT” and ΔPDE2 *S. cerevisiae* strains [4] were kindly provided by Dr. Jeong-Yoon Kim (Chungnam National University, Republic of Korea). The strains were incubated in YEPD liquid medium at 30 °C overnight and different 1/100 made dilutions made with synthetic complete (SC) medium (prepared using 20-g/L glucose, Sigma-Aldrich yeast nitrogen base Y0626 with Sigma-Aldrich yeast synthetic drop-out medium supplement Y1501, used in the manufacturer’s recommended proportions and uracil at 0.1 g/L).

Cultures were fortified with different solutions that were sterilized by filtration: (i) 400-µM RSV, (ii) 400-µM OXY and (iii) 25-µM roflumilast. The cultures were maintained at 30 °C with constant shaking for the entire experiment. Every 2–3 days, 5 µL of these cultures were inoculated with 145 µL of YEPD and left for 24 h at 30 °C in 96-well plates. Abs 600 nm was automatically measured every 15 min (shacking intensity: 3 of 5) using a Synergy HT plate reader (Bio-Tek Instruments, Winooski, VT, USA). Border plates were filled with sterile water to prevent evaporation. The doubling time (δ) and survival (S_n_) were calculated and compared in accordance with the protocol [24].

#### 2.2.6. Data Analysis

The experiments were carried out in triplicate. Graphic representations were made using SigmaPlot (version 10.0) and Microsoft Excel (version 2007). A Michaelis–Menten plot was analyzed using the nonlinear plot available in SigmaPlot (version 10.0). A t-test was applied using Social Science Statistics (https://www.socscistatistics.com/) fixing the significance level at *p* < 0.05. Other mathematical operations were carried out using wxMaxima software (version 12.04.0).

## 3. Results and Discussion

### 3.1. Comparative Sequence Analysis between Human and Yeast PDEs

**Figure 2:**
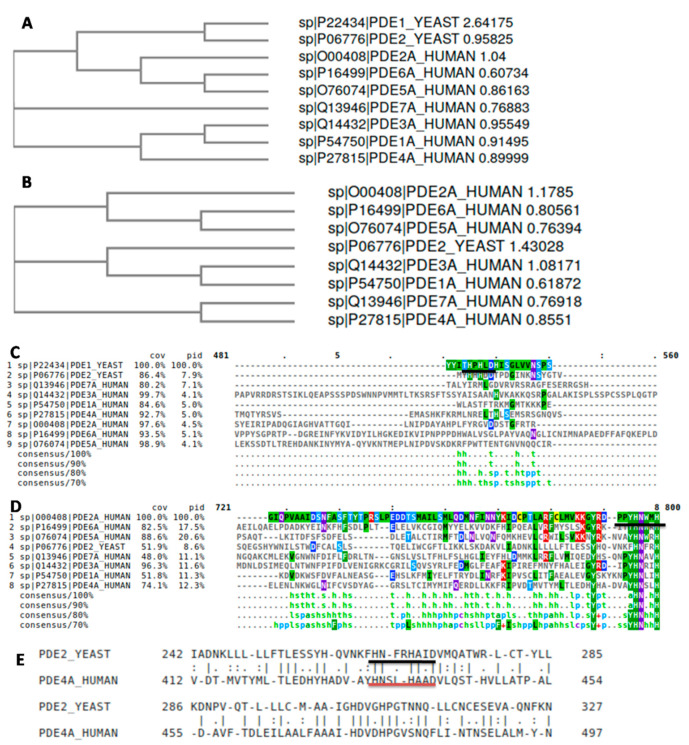
NJ phylogenic tree of all (**A**) human and yeast PDEs and (**B**) without yPDE1; (**C**) detail of MUSCLE multiple alignment for all PDEs; indicated, the yPDE1 active site; (**D**) detail of MUSCLE multiple alignment for all PDEs without yPDE1; indicated, the yPDE2 active site; (**E**) pairwise alignment between yeast yPDE2 and human PDE4A; indicated the active sites.

As mentioned above, RSV has been successfully tested as human PDE inhibitor [18]; furthermore, it was able to inhibit several PDEs. These data allowed us to choose the best yeast PDE to study using multiple sequence alignments (Figure 2). MUSCLE was used to generate the alignment and two different distance trees were constructed, with or without PDE1 (yPDE1, Figure 2A,B, respectively). As can be seen, yPDE1 is quite distant from the rest of the PDEs studied, as demonstrated by its differences of classes (I or II, [12]). Although yeast PDEs form their own branch, the distance can be reduced by removing yPDE1, which suggests some structural differences due to the different functions in yeast metabolism [10]. Perhaps, yPDE2 functions like human PDEs, suggesting some similarity in their respective active sites. Although human PDE isoform 9 is the closest to yPDE2 (Supplementary Materials SM1) according to its sequence, followed by human_PDE4, human_PDE4 was able to revert the heat-shock phenotype, which is characteristic of phosphodiesterase-deficient *S. cerevisiae* [29], suggesting a possible complementary activity.

When the MUSCLE results were checked, the active site of yPDE1 (Figure 2C) was seen to be far from yPDE2 and human PDEs active sites (Figure 2D), which the active His residue and neighbors matched perfectly. Moreover, a pairwise sequence alignment between yPDE2 and human_PDE4A, the aging-related PDE [18], showed 37.9% of similarity. However, the bioactive sites are well conserved (Figure 2E). This, together with the information available in the consulted bibliography suggest a close similarity between the novel competitive inhibitors tested in yPDE2 and their application in human PDE studies. For all the reasons mentioned above, yPDE2 was selected as target enzyme for our study.

### 3.2. yPDE2 Expression and Assay

**Figure 3:**
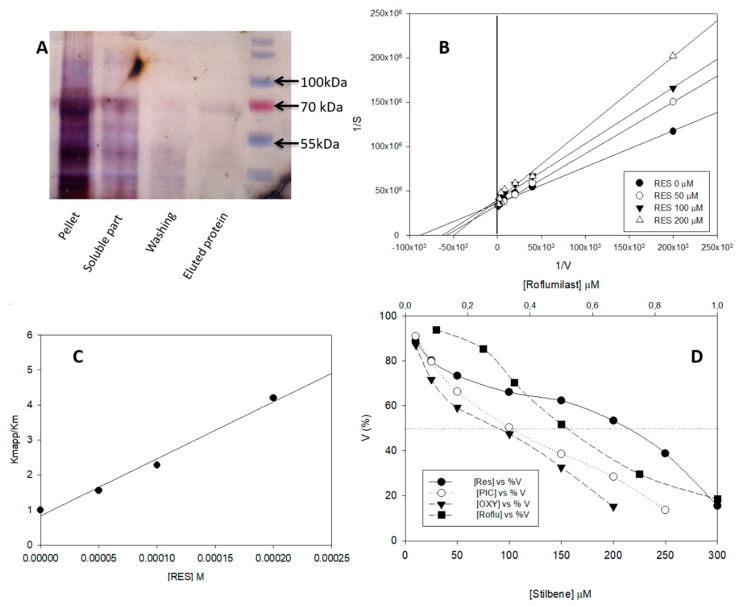
(**A**) SDS-Page silver staining of yPDE2 purification (enzyme MW ≈63 KDa); (**B**) Lineweaver–Burk plot of the effect of different RSV concentrations on yPDE2 activity (sample buffer at 25 °C); (**C**) competitive Michaelis–Menten plot (Equation (3)); (**D**) effect of different inhibitors against yPDE2 activity (sample buffer at 25 °C) using 0.08 μM of enzyme in all cases.

Although yPDE2 has previously been purified and characterized [7,9], this is the first time that the heterologous expression of this enzyme has been considered. *E. coli* was selected because it is easy to manage for protein expression [30]. The available UniProt sequence was used to construct an IPTG-induced vector and Rosetta 2 *E. coli* strain was transformed. Different ranges of inductor, temperatures and other conditions were tested to improve the expression yield. However, by far the greatest part of the protein was always found in the pellet (Figure 3A) and little in the soluble part. Taking this into account, a protocol based on concentration after His-tag purification was followed. While the amount of purified protein was still low, the quantity and purity were considered sufficient to start protein characterization although the authors should perhaps us the term “apparent” parameters.

Our next step was to check the activity of pure yPDE2 and to ascertain whether it is able to convert cAMP into AMP. As intracellular yeast pH values are between 7.5 and 6.4 [9], pH 7 was selected for our enzymatic assays. Figure 3B shows the Lineweaver–Burk plot, with a K_mapp_ = 11.0 ± 0.5 μM, V_maxapp_ = 2.70 × 10^−8^ ± 1.36 × 10^−9^ mols/s/mg and k_catapp_ = 0.001 ± 3 × 10^−5^ s^−1^ (R^2^ ≈ 0.98). This K_mapp_ value is close to the previously reported K_m_ values [7,9] of 1 μM and 0.17 μM respectively at pH 8, for protein purified directly from *S. cerevisiae*, suggesting that our recombinant expression was sufficient to test the inhibition profiles.

### 3.3. Effect of Different Stilbenes and Roflumilast on yPDE2 Activity

In this section, the effect of RSV on yPDE2 activity was tested. The value of K_mapp_ was higher than that of K_mapp_ with small changes observed in V_maxapp_. The results suggested a competitive inhibition, which was also demonstrated using the Lineweaver–Burk plot (Figure 3B) at different RSV concentrations. Interestingly, the same behavior was obtained with human_PDE4 [18]. Taking this into account, Equation (3) was used to construct a plot (Figure 3C, R^2^ > 0.989) with a K_iapp_ value of 63.00 ± 3 µM. The high R^2^ value helped confirm our assumption of competitive behavior.

Several authors [26] use the transformation from I_50_ to K_i_ to analyze comfortably the K_i_, because it easier to obtain. As the structures of RSV, PIC and OXY are quite similar, this approach was followed. Different inhibitor concentrations were incubated at fixed cAMP and enzyme concentrations (Figure 3D) and Equation (4) was used to obtain the K_iapp_ value (Table 1). RSV presented a higher K_iapp_ value than PIC and OXY; indeed, the values of PIC and OXY were not showing any statistical difference (*t*-test, *p* > 0.05). Finally, a common human_PDE4 inhibitor, roflumilast, was tested, which gave the lowest K_iapp_ value (Table 1), perhaps because roflumilast is a specific human_PDE4 inhibitor. To sum up, the following order was obtained for K_iapp_: roflumilast < OXY ≈PIC < RSV.

The following should be mentioned (i) The extra hydroxyl group in the second phenolic ring of stilbenes could affect the inhibition capacities of these compounds depending on the PDE isoforms [31] and (ii) the active site is probably similar because roflumilast was not only able to inhibit yPDE2, but was also the strongest inhibitor tested. These results as a whole suggest that in our in vitro conditions yPDE2 is qualitatively similar to human_PDE4.

### 3.4. Molecular Docking Analysis

**Figure 4:**
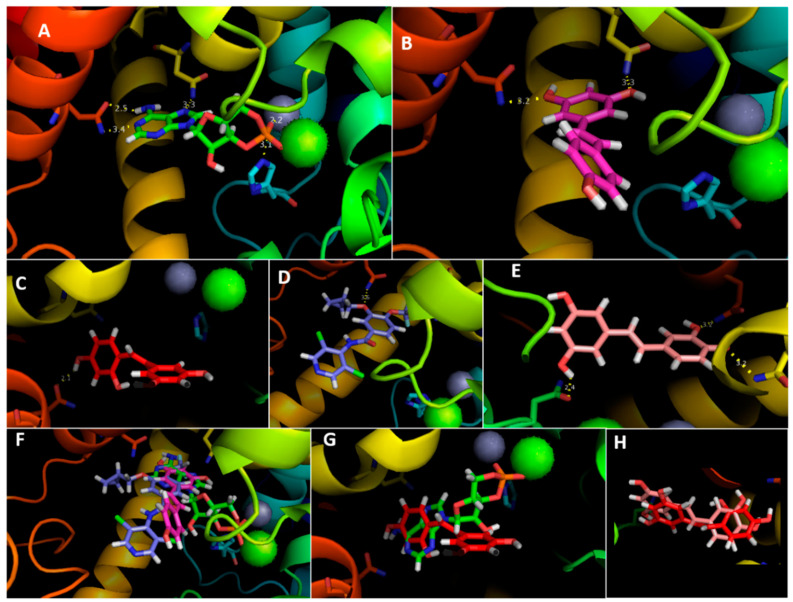
Molecular docking of (**A**) cAMP, (**B**) RSV, (**C**) OXY, (**D**) roflumilast and (**E**) PIC with yPDE2; in yellow the hydrogen bonds. Overlapping cAMP with RSV/roflumilast (**F**) and OXY (**G**) with yPDE2; (**H**) overlapping OXY/PIC with yPDE2.

In order to obtain more information about the inhibition process, molecular docking simulations were carried out. After obtaining a structural model of yPDE2, the interaction with each molecule was simulated. The results (Figure 4 and Table 1) are in perfect agreement with our experimental values. First, the cAMP score was the lowest of all molecules tested, even presenting interactions with His265 (Figure 4A). Moreover, the docking was also supported superimposing the human PDE4D_cAMP crystal (PDB 2PW3), which fitted perfectly (Supplementary Figure S1). Uniprot suggests a His residue as the active site, which agrees with our results. Moreover, this His is conserved in all human PDEs (Figure 2D). On the other hand, cAMP had polar contact with both metal ions (Zn^2+^ and Mg^2+^), which is crucial for its activity; Asn403 and Gln483 also showed a degree of contacts, perhaps to manage the substrate fit.

RSV docking (Figure 4B) interacted with the same residues (Asn403 and Gln483) although not with metal ions or His265, which suggests that both residues are essential for substrate interaction. Indeed, the rest of the inhibitors (Figure 4C–E) interacted with Gln483 at least, reinforcing the importance of this interaction for yPDE2 activity.

If we superimpose the inhibitors on cAMP, there is a strong spatial coincidence (Figure 4F,G), in the nicotine ring of cAMP, a zone that interacts with the Gln483 residue. Although molecular docking suggests that PIC would be better inhibitor than OXY even though they match almost perfectly (Figure 4H), experimental PIC and OXY K_i_ values were similar. Interestingly, the extra hydroxyl group compared with RSV may be responsible for increasing inhibition in RSV, the principal phenolic ring interacts with Gln483 (Figure 4B), but, in OXY/PIC, it is the other ring that does so (Figure 4C,E).

### 3.5. In Vivo Lifespan Studies with S. cerevisiae

**Figure 5:**
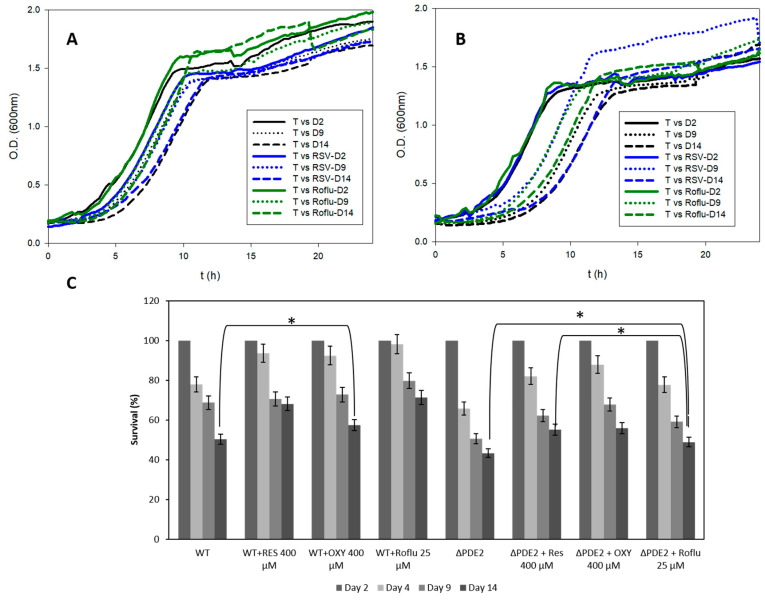
Optical density of maintained cultures at different times (Day 2, 9 and 14) for (**A**) WT and (**B**) ΔPDE2 with or without 400-µM RSV or 25-µM roflumilast; (**C**) Comparative analysis of the survival in all treated cultures. (* means *p* < 0.05 in most relevant results, errors bars represent SD).

We have successfully demonstrated the capacity of stilbenes and roflumilast to inhibit yPDE2 and suggest the most important residues for the interaction. However, the overall objective of this study was to determine the real capacity of these molecules to increase yeast lifespan. For that reason, CSL studies were carried out using two strains, “WT” and yPDE2 KO (ΔPDE2). Among the stilbenes, (a) RSV and (b) OXY were tested at 400 μM. PIC was not tested because OXY and PIC have a similar K_i_, and the meta-OH structure of OXY would be more stable [32,33]; we also tested 25 μM of roflumilast. The concentrations were selected according to the maximum solubility achieved in the cell culture without precipitation (as OXY is less soluble than RSV, OXY solubility was used to fit an equal concentration). Figure 5 summarizes the results obtained. In particular, Figure 5A,B show the effect of the molecules on lifespan, which diminished the increase of δ and t_50_, a sign of their antiaging effect on the cells [24]. As previous results demonstrated, the deletion of yPDE2 decreases the lifespan of the cell [4,16]. However, this pharmacological reversible inhibition seems to increase the lifespan, perhaps any internal yPDE2 up–down regulation [34] or due to the multitasking and different targets of RSV such as Sir2, which also affects lifespan [5,20]. In addition, this is the first time that roflumilast, a common PDE4 inhibitor drug for chronic obstructive pulmonary disease has been studied in lifespan studies although the use of roflumilast to mimic CR has been tested successfully previously [22]. The strains treated with roflumilast were seen to grow faster than WT control or RSV after a delay time. So, it is possible that this drug could extend chronological lifespan via different mechanisms: linking cellular aging to cell cycle regulation [35,36] via cAMP modulation or roflumilast could also modulate yPDE1 as molecular docking result predict (Supplementary Figure S2). Indeed, roflumilast was seen to be able to increase lifespan as RSV in a comparative way, but with different mechanisms [20]. However, these results suggest novel uses of PDE inhibitors.

Figure 5C presents a comparative analysis of all the treatments analyzed. Although at the beginning the three molecules showed around 90% survival (vs. 77% for the control), of the two stilbenes tested, (OXY and RSV), RSV showed better survival at Day 14 (68%) results on the last day, although its K_i_ value was higher than that of OXY (57% at Day 14) and the control (50% at Day 14); perhaps the faster oxidation of OXY in comparison with RSV reduces its effectiveness. Roflumilast showed close values (71% at Day 14) to RSV. In an interesting study [37], RSV decreased the CLS of *S. cerevisiae* when it was administered each 48 h due to the increase in the antioxidant stress. There are several articles on how high-dose antioxidant supplements may be harmful in some cases because they turn pro-oxidant agents [38]. RSV is no exception, depending on its concentration and time of exposure [39,40]. In our study, RSV was administered only at the beginning of the study decreasing the total quantity of RSV (the initial dosage affected the initial growth of *S. cerevisiae*, but the cells were able to recover), leading to it become an antioxidant and lifespan modulator, which may explain our different results.

## 4. Conclusions

This contribution has studied the effect of different molecules (stilbenes and roflumilast) on PDE activity in vitro and in vivo. A phylogenetic tree showed that, although *S. cerevisiae* presents two PDEs, only yPDE2 is close to human PDEs, including the active site. Therefore, this PDE was expressed and a study of the inhibition profile of RSV, OXY, PIC and roflumilast pointed to their competitive behavior with the following K_iaapp_ order: Roflumilast < OXY ≈PIC < RSV. An in silico study was also used to corroborate the results, and the non-covalent interaction suggested important residues for substrate cleavage. Finally, the in vivo lifespan effect was determined with WT and ΔPDE2 strains. The results showed that all molecules tested were able to increase the life expectancy of *S. cerevisiae* strains suggesting novel dietary uses. The tested stilbenes and roflumilast may act as PDE inhibitors; however, it seems that this in vivo activity does not play a crucial role in increase the lifespan of *S. cerevisiae* strains.

## Figures and Tables

**Table 1:** Experimental K_i_ of values predicted docking score for each molecule and hydrogen bond residue distance.

	Inhibitors				Substrate
	Roflu	OXY	PIC	RSV	cAMP
K_iapp_ (µM)	0.30 ± 0.02	46.00 ± 4.00	50.00 ± 5.00	63.00 ± 3.00	–
Docking score	−8.01	−6.34	−6.93	−6.22	−14.36
Residue distance (Å)					
Gln 483	3.6	2.1	3.2	3.2	2.5 & 3.4
Asn 403	–	–	3.2	3.3	3.3
His 265	–	–	–	–	3.1
Asn 310	–	–	2.4	–	–
Mg	–	–	–	–	1.0
Zn	–	–	–	–	2.2

## Supplementary Materials

The following are available online at https://www.mdpi.com/1424-8247/13/9/225/s1, SM1: Blast results of yPDE2 analyzing the human database; SM2: Needle report about human AMPK and Yeast Snf1 kinase subunit 1; SM3: Needle report about human AMPK and Yeast Snf1 kinase subunit 2; Figure S1: Overlapping of human PDE4D (pink) and cAMP (red) from PDB 2PW3 with our protein predicted model (blue) and docked cAMP (green); Figure S2: Overlapping of yPDE1 4OJV crystal and cGMP (red) and docked Roflumilast (green)
