# Supplementary material for: Characterization of Resveratrol, Oxyresveratrol, Piceatannol and Roflumilast as Modulators of Phosphodiesterase Activity. Study of Yeast Lifespan
Source: Pharmaceuticals (Basel). 2020 Aug 30;13(9):225. doi: 10.3390/ph13090225 (PMC7559934; doi:10.3390/ph13090225)

**SUPPLEMENTARY MATERIAL**

*Supplementary material 1. Blast results of yPDE2 analyzing the human database.*

*Supplementary material 2 and 3: Needle report about human AMPK and Yeast Snf1 kinase subunits.*

*Supplementary Figure 1. Overlapping of human PDE4D (pink) and cAMP (red) from PDB 2PW3 with our protein predicted model (blue) and docked cAMP (green).*


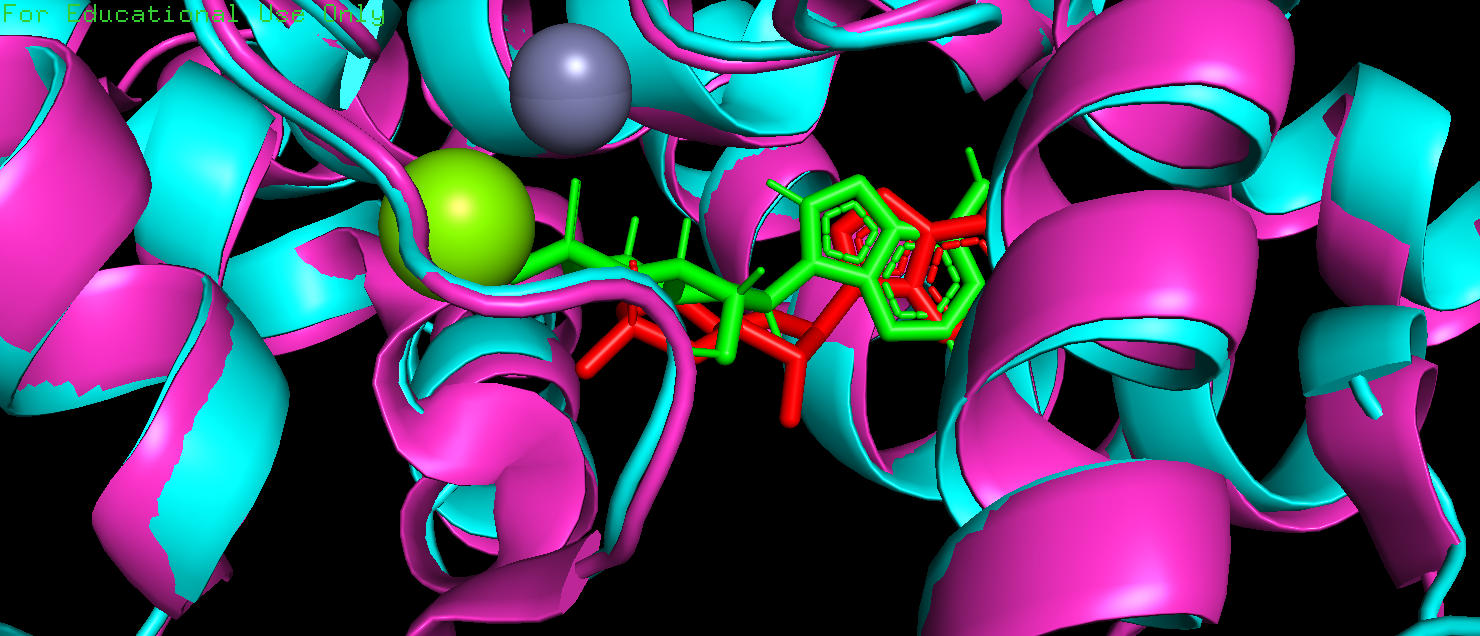


*Supplementary Figure 2. Overlapping of yPDE1 4OJV crystal and cGMP (red) and docked Roflumilast (green).*


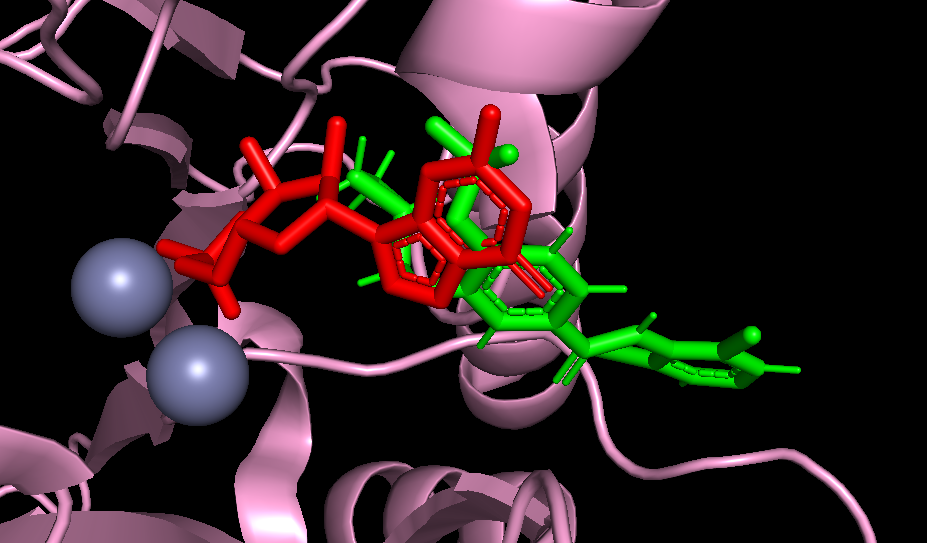

Supplement: Supplementary file 1 [file pharmaceuticals-13-00225-s001.zip › Supplementary v2.docx]
